# Evaluation of Anti-Inflammatory Activity of the New Cardiotonic Steroid γ-Benzylidene Digoxin 8 (BD-8) in Mice

**DOI:** 10.3390/cells13181568

**Published:** 2024-09-18

**Authors:** Davi Azevedo Ferreira, Anna Beatriz Araujo Medeiros, Mariana Mendonça Soares, Éssia de Almeida Lima, Gabriela Carolina Santos Lima de Oliveira, Mateus Bernardo da Silva Leite, Matheus Vieira Machado, José Augusto Ferreira Perez Villar, Leandro Augusto Barbosa, Cristoforo Scavone, Marcelo Tigre Moura, Sandra Rodrigues-Mascarenhas

**Affiliations:** 1 Laboratory of Immunobiotechnology, Biotechnology Center, Federal University of Paraiba, João Pessoa 58.051-900, PB, Brazil; daviazevedoferreira@ltf.ufpb.br (D.A.F.); annabbeatrizz@gmail.com (A.B.A.M.); marimsoaress@gmail.com (M.M.S.); essia_almeida@hotmail.com (É.d.A.L.); gabrielacarolina10@gmail.com (G.C.S.L.d.O.); mateusvidamb@gmail.com (M.B.d.S.L.); 2 Laboratory of Cellular Biochemistry, Campus Centro-Oeste Dona Lindú, Federal University of São João del-Rei, Divinópolis 35.501-296, MG, Brazil; matheusmg15@gmail.com (M.V.M.); zevillar@ufsj.edu.br (J.A.F.P.V.); lbarbosa.ufsj@gmail.com (L.A.B.); 3 Laboratory of Neuropharmacology Research, Department of Pharmacology, Institute of Biomedical Sciences ICB-1, University of São Paulo, São Paulo 05.508-900, SP, Brazil; cristoforo.scavone@gmail.com; 4 Laboratory of Cellular Reprogramming, Biotechnology Center, Federal University of Paraiba, João Pessoa 58.051-900, PB, Brazil; marcelotmoura@gmail.com

**Keywords:** macrophages, phagocytosis, IL-1β, NF-κB, ERK, p38, inflammation

## Abstract

Cardiotonic steroids are known to bind to Na+/K+-ATPase and regulate several biological processes, including the immune response. The synthetic cardiotonic steroid γ-Benzylidene Digoxin 8 (BD-8) is emerging as a promising immunomodulatory molecule, although it has remained largely unexplored. Therefore, we tested the immunomodulatory potential of BD-8 both in vitro and in vivo. Hence, primary mouse macrophages were incubated with combinations of BD-8 and the pro-inflammatory fungal protein zymosan (ZYM). Nitric oxide (NO) production was determined by Griess reagent and cytokines production was assessed by enzyme-linked immunosorbent assay. Inducible nitric oxide synthase (iNOS), reactive oxygen species (ROS), p-nuclear factor kappa B p65 (NF-κB p65), p-extracellular signal-regulated kinase (p-ERK), and p-p38 were evaluated by flow cytometry. Macrophages exposed to BD-8 displayed reduced phagocytic activity, NO levels, and production of the proinflammatory cytokine IL-1β induced by ZYM. Furthermore, BD-8 diminished the expression of iNOS and phosphorylation of NF-κB p65, ERK, and p38. Additionally, BD-8 exhibited anti-inflammatory capacity in vivo in a carrageenan-induced mouse paw edema model. Taken together, these findings demonstrate the anti-inflammatory activity of BD-8 and further reinforce the potential of cardiotonic steroids and their derivatives as immunomodulatory molecules.

## 1. Introduction

Inflammation is a physiologically relevant process for host defense, tissue repair, and homeostasis. However, persistent inflammation may evolve into a chronic process that ultimately leads to tissue damage. [1,2]. Under such context, it remains paramount to block the inflammatory response by reducing the production of pro-inflammatory factors/mediators, or alternatively, by stimulating anti-inflammatory ones [3]. Several cell types participate in the inflammatory response, with macrophages playing a key role. Macrophages originate from monocytes circulating in the bloodstream and differentiate upon reaching the site of inflammation [4]. Tissue-resident macrophages typically produce low levels of inflammatory mediators but significantly increase production when exposed to pro-inflammatory signals, such as fungal gene products like zymosan (ZYM) [5].

Cardiotonic steroids, such as ouabain, marinobufagenin and digoxin, bind to Na^+^/K^+^-ATPase and thus control several biological activities, including anti-inflammatory activity [6,7]. These molecules may counteract inflammatory parameters such as cell migration, vascular permeability, and proinflammatory cytokines [8,9,10,11,12,13]. Our group has demonstrated the anti-inflammatory role of ouabain by showing its ability to lower pro-inflammatory cytokine levels [7] and inhibit the activation of mitogen-activated protein kinase (MAPK) p38/nuclear and factor (NF)-κB signaling [14]. Furthermore, ouabain also negatively modulates several aspects of the allergic asthma response [15]. Our group has also demonstrated the anti-inflammatory role of marinobufagenin by showing its ability to lower levels of the pro-inflammatory cytokines IL-1β and IL-6, as well as reduce neutrophil migration in vivo. Additionally, it decreased nitric oxide and cytokines IL-1β, IL-6, and TNF-α in vitro, without interfering with the expression of surface molecules (TLR2, CD69) and MAPK p38 [16]. In light of these findings, the proposed mechanism of digoxin’s anti-inflammatory role inhibited Th0 cell differentiation into Th17 cells [17]. Furthermore, other proposed mechanisms for digoxin’s anti-inflammatory role include the inhibition of Nuclear Factor kappa B (NF-κB), an important central regulator of inflammation [17,18,19].

Derivatives of endogenous cardiotonic steroids have shown promising results. A digoxin derivative named BD-21 (γ-benzylidene digoxin 21) held unique chemical traits from other cardiotonic steroids although it showed anti-inflammatory activity, significantly inhibiting paw edema, decreasing the expression of iNOS and the levels of TNF-α [20]. These findings demonstrate the potential of new cardiotonic steroids as immunomodulatory molecules. Another digoxin derivative, BD-15 (γ-benzylidene digoxin 15), has shown a neuroprotective effect by preventing H_2_O_2_ production, lipid peroxidation, and other factors related to oxidative stress damage [21]. Like digoxin, BD-8 and BD-15 inhibit Na+/K+-ATPase function, but they also can interfere with other cell signaling pathways. Preliminary studies indicate that these molecules influence cellular proliferation and induce apoptosis in cancer cells, thus suggesting a multifaceted mechanism of action [16]. Moreover, these synthetic cardiotonic steroids are also studied for their anticancer effects. In preclinical models, these molecules have been shown to inhibit the growth of cancer cells and induce apoptosis. BD-21 and BD-15, in particular, have been highlighted for their potential in therapies against aggressive cancers, such as melanoma and lung cancer [22,23,24].

These findings were also observed in the context of inflammatory processes, further reinforcing the potential use of benzylidene digoxin derivatives. BD-8 is a novel and unexplored benzylidene digoxin derivative, synthesized by our research group with the addition of an ether group to its structure. This new cardiotonic steroid, analogous to ouabain, demonstrated selective activation of the α2 isoform of Na^+^/K^+^-ATPase in a Sf9 heterologous expression system [25]. In tissue immune responses, macrophages play a pivotal role by recognizing molecular patterns and responding through phagocytosis and cytokine release. However, the role of BD-8 on activated macrophages has not yet been investigated. Therefore, this work aimed to analyze the immunomodulatory activity of BD-8 using in silico, in vitro, and in vivo experimental approaches.

## 2. Materials and Methods

### 2.1. Synthesis of γ-Benzylidene Digoxin 8 (BD-8)

BD-8 was synthesized by chemical modification of the digoxin molecule, carried out by the Laboratory of Organic Synthesis and Nanostructure of the Federal University of São João del-Rei, Minas Gerais, Brazil. The new cardiotonic steroids BD-8 is within cardenolides groups, with an additional group aromatic with six members, and BD-8 was synthesized by adding one ether group to the group aromatic with six members [25]. A stock solution of BD-8 (5.0 mg/mL) was prepared with dimethyl sulfoxide (DMSO) (Sigma Chemical Co. St. Louis, MO, USA) and freshly diluted in RPMI-1640 medium (Invitrogen-Gibco, Grand Island, NY, USA) before all experiments; the final DMSO concentration never exceeded 0.08%. In the control condition, only the RPMI-1640 medium was used.

### 2.2. In Silico Analysis

The in silico analyses used BD-8, BD-21, ouabain, and digoxin (Table S1). The SMILES annotation of each compound was retrieved from Pubchem (digoxin: CID 2,724,385 and Ouabain: CID 439501) or annotated manually (BD-8 and BD-21). Molecule toxicity and pharmacokinetics were carried out with ProTox3.0 [26] and Deep-PK [27]. Cellular viability was predicted with CLC-Pred2.0 with default parameters [28]. The prediction of gene expression changes and protein levels was based on the DIGEP-Pred tool using a Pa > 0.8 cutoff [29]. Functional enrichment was done with g: Profiler for *Homo sapiens* (Human) (https://biit.cs.ut.ee/gprofiler/gost accessed on 3 June 2024). Pathway analysis and cellular processes were predicted with the tool PASS with a cutoff of Pa > 0.7 [30,31].

### 2.3. Animals

All protocols adopted in this study were approved by the Institutional Ethics Committee (protocol: 5274120522; ID: 017192). Female Swiss albino mice (6−8 weeks) were provided by the Prof. Thomas George Animal House of the Federal University of Paraiba. The animals were kept under standard laboratory conditions on a constant 12 h light/dark cycle with controlled temperature (21 ± 2 °C). Food and water were provided ad libitum.

### 2.4. Macrophage Isolation

The in vitro model of peritoneal macrophages was performed as previously described [32,33,34]. The peritoneal exudate was briefly elicited with an intraperitoneal (i.p.) injection of 2.0 mL of 4% thioglycollate medium (Sigma Chemical Co. St. Louis, MO, USA). Four days later, the animals were euthanized, and the peritoneal cavity was washed with 5.0 mL of cold phosphate-buffered saline (PBS), followed by fluid collection. The cell suspension obtained was centrifuged for 5 min at 300 g (4 °C). The supernatant was discarded, and the pellet was suspended in 2.0 mL of complete RPMI-1640 medium supplemented with 10% fetal bovine serum (Invitrogen-Gibco Grand Island, NY, USA), 100 units/mL penicillin (Invitrogen-Gibco Grand Island, NY, USA), 0.002 g/mL sodium bicarbonate, and 100 µg/mL streptomycin (Invitrogen-Gibco Grand Island, NY, USA). Macrophages were enriched by adherence to the plastic Petri dishes. Viable cells were seeded in flat-bottom 96-well plates (2.0 ×10^5^ cells/well in a final volume of 200 µL) and incubated for 2 h (5% CO_2_ at 37 °C). Non-adherent cells were washed off and discarded. The adherent cells were stimulated with ZYM (Sigma Chemical Co. St. Louis, MO, USA) (0.2 mg/mL) and treated with different BD-8 concentrations (1 nM, 10 nM, 100 nM, 1 µM, 10 µM, and 100 µM). After 24 h of incubation, the macrophages were used for cell viability assay, and the culture supernatant was collected to determine nitric oxide and cytokine production. Six (6) animals were used per experiment, with 3 biological replicates.

### 2.5. Macrophage Viability

Cell viability was estimated by the MTT assay [35]. For that, 100 µL of complete RPMI-1640 medium containing 10 µL of MTT solution (Sigma Chemical Co. St. Louis, MO, USA) (5 mg/mL MTT in PBS) was added to each well. After 4 h of incubation, the MTT-containing medium was discarded, and the precipitate was solubilized in 100 µL DMSO. Optical density was read at 570 nm.

### 2.6. Nitric Oxide Production

Nitric oxide production was indirectly estimated by measurement of nitrite, a major stable product of nitric oxide, using Griess solution [36]. Briefly, 50 µL of the culture supernatant was mixed with an equal amount of Griess solution at room temperature for 10 min. Optical density was read at 540 nm, and sodium nitrite was used for the standard curve.

### 2.7. Quantification of Cytokine Production

According to the manufacturer’s instructions (Invitrogen-Gibco Grand Island, NY, USA), IL-1β, IL-6, IL-10, and TNF-α levels in the culture supernatant were determined by sandwich enzyme-linked immunosorbent assay. Optical density was read at 450 nm.

### 2.8. Zymosan Phagocytosis Assay

Peritoneal macrophages were seeded on coverslips in 24-well plates at a density of 5.0 × 10^5^. The plates were incubated for two hours (atmosphere of 5% CO_2_ at 37 °C) to allow cell adhesion and further incubation with concentrations of BD-8 10 μM for 24 h. Thereafter, the contents of the wells were removed, 0.2 mg/mL ZYM was added, and the plate was incubated for 40 min. The wells were again washed with PBS, and 300 μL of 4% paraformaldehyde (4% PFA) was added for overnight fixation. Finally, the coverslips were stained using the rapid panoptic method, mounted on microscope slides with the aid of Canada Balsam, and viewed under an optical microscope with a 100× immersion objective.

The percentage of phagocytosis was estimated by counting phagocytosed zymosan particles in 100 macrophages, with macrophages with increased phagocytic activity considered to have at least three internalized zymosan particles [37].

### 2.9. Effect of BD-8 on Macrophages Functions and Its Mechanism of Action

After treatment with BD-8 (10 µM) for 24 h, phagocytosis of zymosan particles was assessed by two methods: optical microscopy analysis and flow cytometry accordingly and pHrodo™ Red Zymosan BioParticles™ Conjugate for Phagocytosis Kit (P35364 Invitrogen™), respectively, after 40 min of zymosan stimulus. Similarly, ROS production was assessed accordingly CellROX™ Green Flow Cytometry Assay Kit (C10492 Invitrogen™). Furthermore, macrophages were cultured in flat-bottom 6-well plates (1.5 × 10^6^ cells/well) and incubated for 2 h. Then, the cells were treated with 10 µM BD-8 and stimulated with 0.2 mg/mL ZYM. After 24 h of incubation, cells were collected and placed in U-bottom 96-well plates. Then, cells were blocked to prevent non-specific Fc-mediated interactions using anti-CD16/CD32 and labeled separately with anti-iNOS, anti-NF-κB p65, anti-p-ERK, and anti-p-p38 (Thermo Fisher Scientific-Waltham, MA, USA), according to the manufacturer’s instructions. Finally, cells were suspended in PBS and evaluated using a flow cytometer. A minimum of 10,000 events was acquired for each sample.

### 2.10. Model of Paw Edema

Mice were randomly divided into nine groups and five animals per group (*n* = 5). To analyze the anti-inflammatory activity of BD-8 in vivo, mice received subcutaneous injections in the plantar surface [38] of 2.5% carrageenan (Sigma Chemical Co. St. Louis, MO, USA) solubilized in 20 μL of PBS in the right (ipsilateral) hindpaw and 20 μL of PBS in the left (contralateral) hindpaw. The mice were treated 1 h before the intraplantar injection of carrageenan with injection i.p. of BD-8 in different dose tests (1.12 mg/kg, 0.56 mg/kg and 0.28 mg/kg) as previously tested with ouabain (0.56 mg/kg) [7,39], testing with BD-8 the double dose (1.12 mg/kg) and half dose (0.28 mg/kg) and the standard drug indomethacin (10 mg/kg). After the treatment, mice were challenged with carrageenan (2.5%), and the quantified paw thickness was measured before (0 h) and at intervals of 1, 2, 3, 4, and 6 h. The measurement was made by the difference between the right hindpaw and the left hindpaw [40]. Hindpaw edema was measured with a digital vernier caliper.

### 2.11. Histological Analysis

After 6 h of carrageenan induced edema, paw samples were taken for histological examination [41]. Paws were removed, fixed in the same solution, descaled and embedded in paraffin. Five-micron-thick sections of paraffin-embedded paws were stained with hematoxylin-eosin (HE) according to standard protocols. Slides were examined under light microscopy (Motic BA 410), and digital photographs were captured with a Moticam 5.0 MP camera.

### 2.12. Statistical Analysis

Flow cytometry data were analyzed using FlowJo^®^ X software version 10.6.2. All data were expressed as mean ± standard error of the mean (SEM) and analyzed using GraphPad Prism^®^ software version 10.0.0 using a one-way analysis of variance followed by Tukey’s test for multiple comparisons. The results were considered statistically significant when *p* < 0.05.

## 3. Results

### 3.1. In Silico Analyses

The prediction accuracy scores of newly synthesized molecules BD-8 and BD-21 were lower (67.38% and 68.07%, respectively) than digoxin and ouabain (100%). Moreover, BD-8 and BD-21 were more similar to digoxin than ouabain, as expected by their derivation from digoxin (Table S2). Both BD-8 and BD-21 were predicted to be safer than digoxin and ouabain in an oral toxicity prediction (predicted toxicity class 2 vs. class 1 of digoxin compared to ouabain), and the former molecules displayed a higher lethal dose than the later ones (8.0 mg/kg vs. 5.0 mg/kg, respectively).

**Figure 1:**
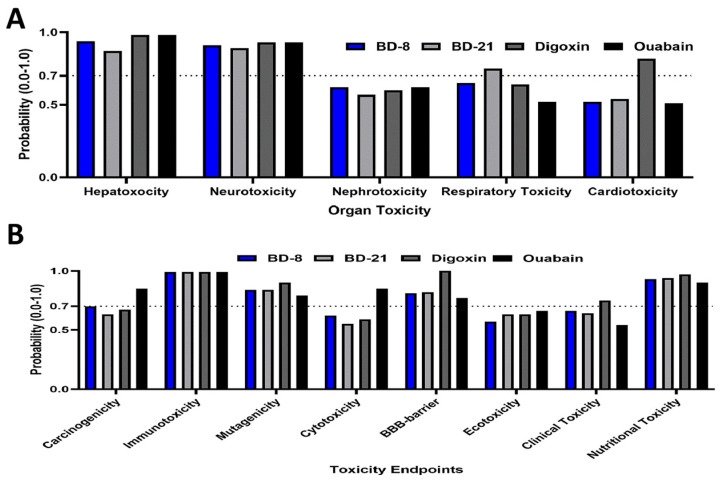
In silico toxicity prediction of BD-8 and other cardiotonic molecules. (**A**) Probability of organ toxicity determined by ProTox3.0. (**B**) Probability of toxicity endpoints determined by ProTox3.0. The dashed lines represent the 0.70 probability cutoffs.

The BD-8 molecule did not display hepatotoxicity and neurotoxicity potentials in a similar fashion to BD-21, digoxin, and ouabain (Figure 1A). Moreover, BD-21 was pre-dicted to cause respiratory toxicity (0.75), and digoxin was safe from a cardiotoxic stand-point (Figure 1A). Further predictions on toxicity endpoints suggested that BD-8 would be inactive as a carcinogenic, mutagenic, and impermeable through the blood–brain barrier (Figure 1B), as predicted for BD-21, digoxin, and ouabain. All four molecules were predicted to be active for immunotoxicity (0.99) and nutritional toxicity (0.90−0.97). There was also a strong overlap of signaling pathways potentially activated by such compounds (Table S3). Similar to BD-21, digoxin, and ouabain, BD-8 was expected to have similar ab-sorption, distribution, metabolism, and half-life (Table S4). Most predictions on toxicity were similar between cardiotonic molecules, although BD-8 was safer under some con-texts, such as rat chronic oral toxicity and respiratory disease (Table S4).

**Figure 2:**
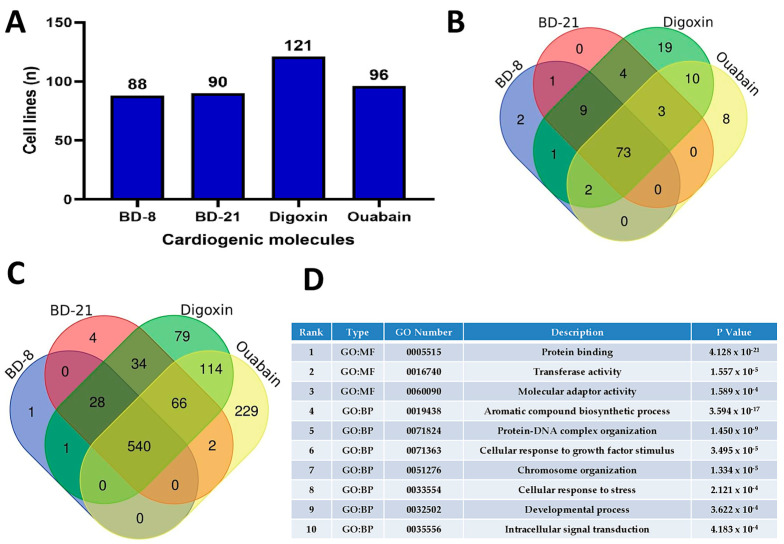
In silico prediction of cytotoxicity and gene expression changes by BD-8 and other cardiotonic molecules. (**A**) Number of tumor and non-tumor cell lines with predicted cytotoxicity scores for BD-8, BD-21, digoxin, and ouabain using CLC-pred2.0. (**B**) Venn diagram of shared cell lines predicted for cytotoxicity using CLC-pred2.0. (**C**) Venn diagram displaying shared gene expression changes predicted in human cell lines MCF7 and VCAP by in silico treatment with BD-8, BD-21, digoxin, and ouabain using DIGEP-Pred. (**D**) Functional enrichment (gene ontology–GO) with shared gene expression among all four cardiotonic molecules (540 gene transcripts) using g: Profiler. The ranking was based on the adjusted P values. BD: γ-benzylidene digoxin derivate. BP: biological process. MF: molecular function.

Multiple cell lines were predicted for cytotoxicity by BD-8 and other cardiotonic molecules (Figure 2A). A total of 132 cell lines were modeled for the four molecules, of which 88 were done for BD-8. From the top 10 cell lines with greater potential for compound activity within cells (Table S5), six lines were shared among the four molecules, nine lines were shared between BD-8 and BD-21, seven between BD-8 and digoxin, and six between BD-8 and ouabain (Figure 2B). There was a substantial overlap within gene expression profiles after in silico treatment of tumor cell lines with cardiotonic molecules (Figure 2C), and only one gene specifically induced by BD-8 (*Slc2a4*, member of the solute carrier family 2–facilitated glucose transporter). Digoxin and ouabain had stronger molecule-specific effects on gene expression profiles (Figure 2C). Functional enrichment showed that molecules have a strong role in gene regulation, cellular response, and signal transduction (Figure 2D). Alternatively, we predicted potential cellular pathways modulated by these molecules [30,31]. Cellular pathways predicted in silico were highly similar between molecules (Table S6), thus including potential as a cardiotonic, antineoplastic, respiratory analeptic, and apoptosis agonist.

### 3.2. Results In Vitro

#### 3.2.1. Effect of BD-8 on Macrophage Viability

**Figure 3:**
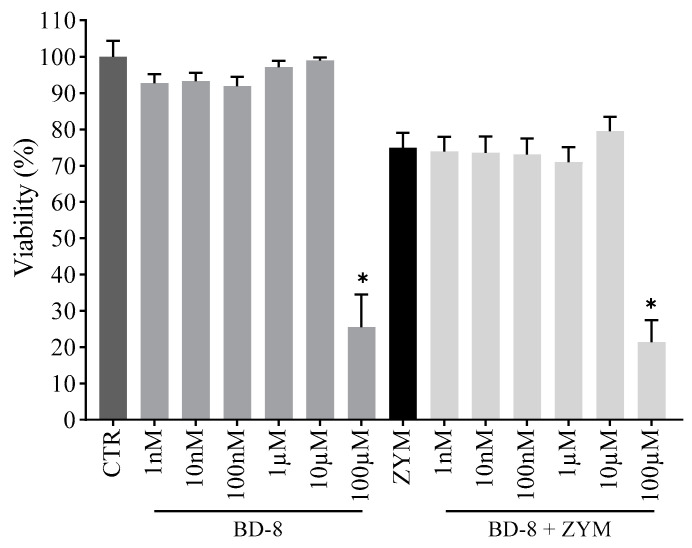
Effect of BD-8 on the viability of murine peritoneal macrophages. Murine peritoneal macrophages were treated with varying BD-8 concentrations (1 nM, 10 nM, 100 nM, 1 µM, 10 µM, and 100 µM) in the absence or presence of 0.2 mg/mL ZYM. After 24 h, the cell viability was assessed by MTT assay. Results were expressed as mean ± SEM and analyzed using GraphPad Prism^®^ software version 10.0.0 using one-way analysis of variance followed by Tukey’s test for multiple comparisons. *—(*p* = 0.05). CTR: Control; ZYM: Zymosan.

As shown in Figure 3, cell viability was not affected by BD-8 at concentrations up to 10 µM in the absence or presence of ZYM when compared to the control group. On the other hand, BD-8 at the highest concentration (100 µM) reduced cell viability and was not used in the following assays.

#### 3.2.2. Effect of BD-8 on Nitric Oxide Production

**Figure 4:**
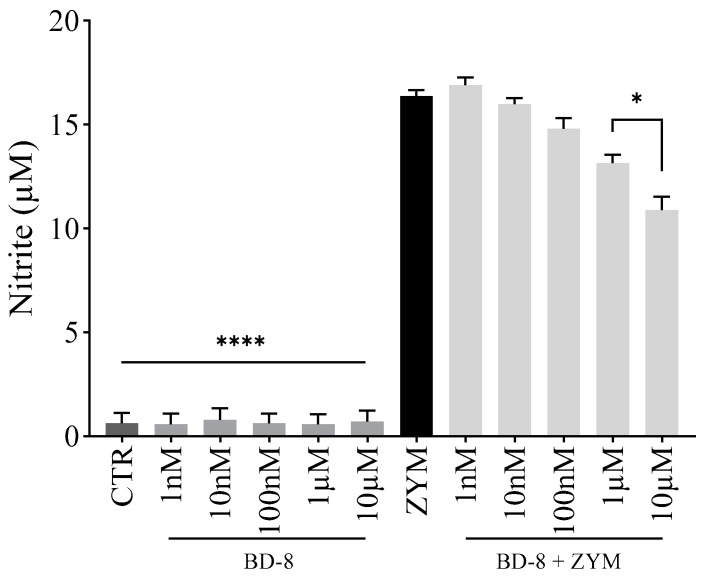
Effect of BD-8 on nitric oxide production. Murine peritoneal macrophages were treated with varying BD-8 concentrations (1 nM, 10 nM, 100 nM, 1 µM, and 10 µM) in the absence or presence of 0.2 mg/mL ZYM. After 24 h, the nitric oxide production was determined by Griess reagent. Results were expressed as mean ± SEM and analyzed using GraphPad Prism^®^ software version 10.0.0 using one-way analysis of variance followed by Tukey’s test for multiple comparisons. ****—(*p* < 0.0001); *—(*p* = 0.05). CTR: Control; ZYM: Zymosan.

As expected, ZYM-stimulated peritoneal macrophages showed increased NO levels (94.7%) compared to the control group (Figure 4). BD-8 (1 and 10 μM) treatment reduced NO levels by 19% and 32%, respectively. However, other concentrations did not change NO levels.

#### 3.2.3. Effect of BD-8 on Cytokine Production

**Figure 5:**
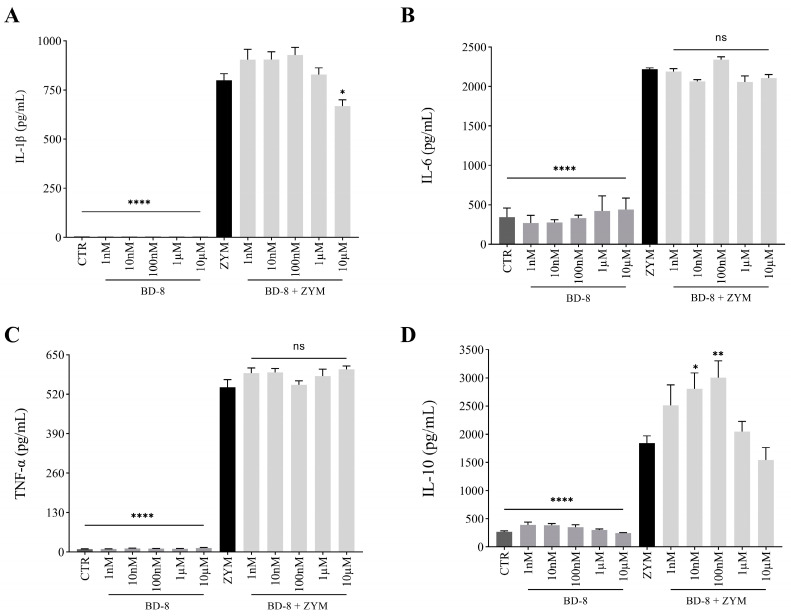
Effect of BD-8 on cytokine production. Murine peritoneal macrophages were treated with varying BD-8 concentrations (1 nM, 10 nM, 100 nM, 1 µM, and 10 µM) in the absence or presence of 0.2 mg/mL ZYM. After 24 h, the IL-1β, IL-6, IL-10, and TNF-α release were determined by enzyme-linked immunosorbent assay. (**A**) production IL-1β; (**B**) production IL-6; (**C**) production TNF-α; (**D**) IL-10. Results were expressed as mean ± SEM and analyzed using GraphPad Prism^®^ software version 10.0.0 using one-way analysis of variance followed by Tukey’s test for multiple comparisons. ****—(*p* < 0.0001); **—(*p* = 0.01); *—(*p* = 0.05). CTR: Control; ZYM: Zymosan; ns: not significant.

Peritoneal macrophages stimulated with ZYM showed an increased production of pro-inflammatory (IL-1β, IL-6, and TNF-α) and anti-inflammatory (IL-10) cytokines compared to the control group. Treatment with 10 µM BD-8 reduced IL-1β levels (12%; *p* = 0.03), whereas the IL-6 and TNF-α were unaffected. Furthermore, BD-8 increased IL-10 production, with maximal response at 100 nM (39.6%; *p* = 0.0002) (Figure 5A−D). The highest concentration of BD-8 demonstrated the greatest NO and IL-1β reduction, being the main concentration used in the other experiments.

#### 3.2.4. Effect of BD-8 on Phagocytosis of ZYM Particles

**Figure 6:**
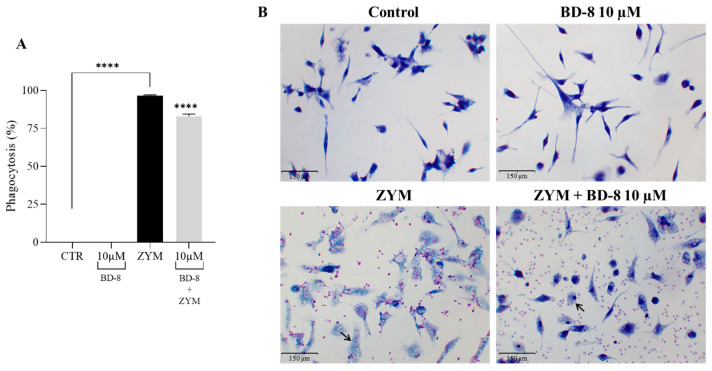
Effect of BD-8 on phagocytosis of ZYM particles. Murine peritoneal macrophages were treated with 10 µM BD-8. After 24 h of incubation, the cells were stimulated with 0.2 mg/mL ZYM (**B**) representative phagocytosis ZYM particles in and incubated again for 40 min and the phagocytosis was evaluated. (**A**) Results were expressed as mean ± SEM and analyzed using GraphPad Prism^®^ software version 10.0.0 using one-way analysis of variance followed by Tukey’s test for multiple comparisons. ****—(*p* < 0.0001); Black arrows–phagocytosed ZYM particles.

The phagocytosis assay revealed an increase of the phagocytic activity of macrophages in the ZYM group compared to the control group. On the other hand, the treatment with 10 µM BD-8 reduced the phagocytosis of ZYM particles by 13.5% (Figure 6A). The biological effect can be observed below (Figure 6B).

#### 3.2.5. Effect of BD-8 on Phagocytosis of Fluorescent Red-ZYM Particles

**Figure 7:**
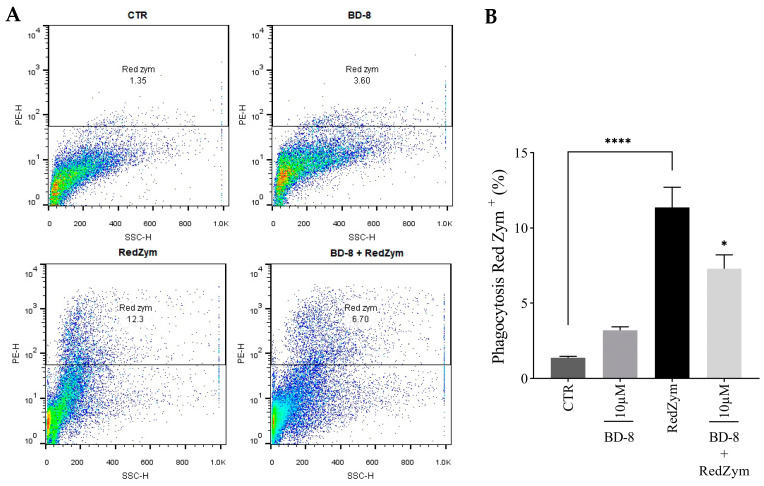
Effect of BD-8 on phagocytosis. Murine peritoneal macrophages were treated with 10 µM BD-8. After 24 h of incubation, the cells were stimulated with 1:10 red ZYM (representative fluorescence dot plots (**A**) and incubated again for 40 min and the phagocytosis was evaluated. Flow cytometry data were analyzed using FlowJo^®^ X software version 10.6.2. (**B**) Results were expressed as mean ± SEM and analyzed using GraphPad Prism^®^ software version 10.0.0 using one-way analysis of variance followed by Tukey’s test for multiple comparisons. ****—(*p* < 0.0001); *—(*p* = 0.05). CTR: Control; RedZym: Red ZYM.

The results revealed that 10 µM BD-8 reduced the phagocytosis of red ZYM particles by 36% (Figure 7A,B).

#### 3.2.6. Effect of BD-8 on ROS Production, iNOS and COX-2 Expression, and NF-κB p65, MAPK–ERK/p38, Akt/mTOR, and Src Phosphorylation

**Figure 8:**
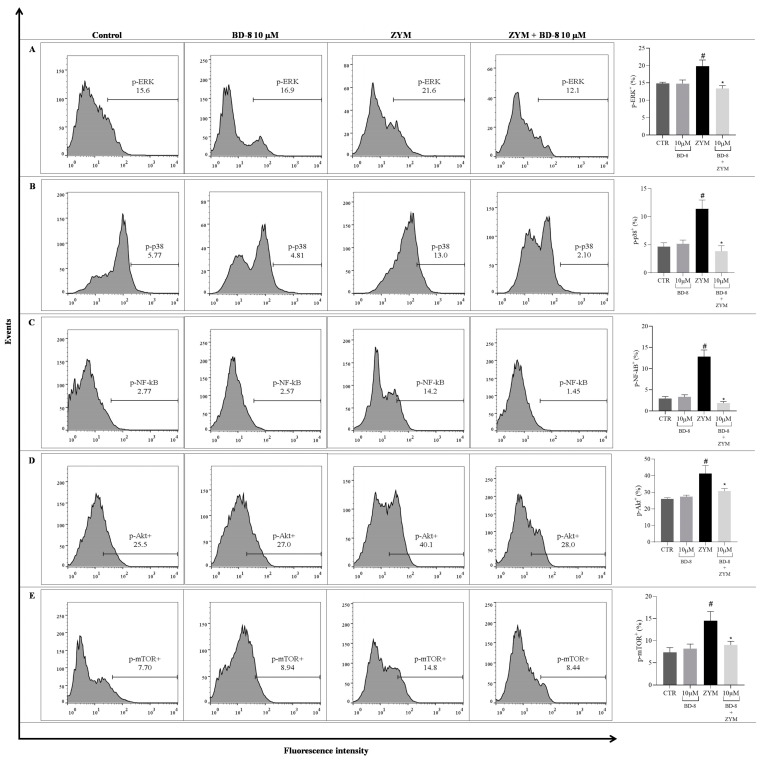

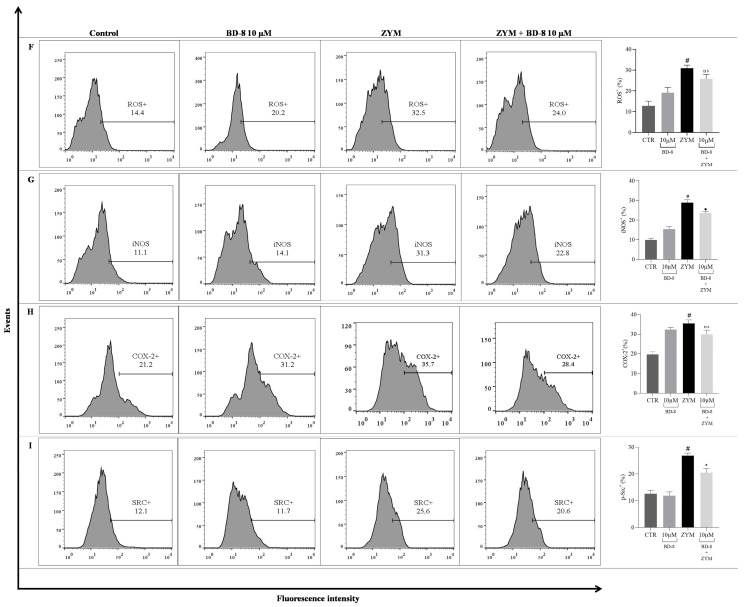
Effects of BD-8 on intracellular molecules associated with inflammatory processes and histograms. Peritoneal macrophages were stimulated with 0.2 mg/mL ZYM and treated with 10 µM BD-8. The cells were evaluated by flow cytometry and marked with specific antibodies for intracellular molecules. (**A**) p-ERK+; (**B**) p-p38+; (**C**) p-NF- κB+; (**D**) p-Akt+; (**E**) p-mTOR+; (**F**) ROS+; (**G**) iNOS+; (**H**) COX-2+; (**I**) Src+. The numerical data are presented as mean ± SEM with an average of *n* = 5 and were analyzed using one-way analysis of variance (ANOVA), followed by Tukey’s post-test. # *p* < 0.05, significant in relation to the control group; * *p* < 0.05, significant in relation to the ZYM group. CTR: Control; ZYM: Zymosan; ns: not significant.

Stimulation with ZYM increased the expression of iNOS (65.4%; *p* < 0.0001), COX-2 (44.5%; *p* = 0.001), ROS levels (58.9%; *p* < 0.0001), and phosphorylation of p38 (58.8%; *p* = 0.0006), ERK (25.2%; *p* = 0.04), NF-κB p65 (76.5%; *p* < 0.0001), Akt (37%; *p* = 0.001), mTOR (49.7%; *p* = 0.008), and Src (53%; *p* < 0.0001) compared to the control group. The group treated with BD-8 at a concentration 10 µM showed reduced expression of the iNOS enzyme (18.34%; *p* = 0.0412), as well as phosphorylation of p38 (66.1%; *p* = 0.0002), ERK (32.7%; *p* = 0.0128), NF-κB p65 (85.8%; *p* < 0.0001), Akt (26%; *p* = 0.03), mTOR (38%; *p* = 0.03), and Src (24%; *p* < 0.0001). However, BD-8 did not modulate ROS and COX-2 (Figure 8).

### 3.3. Results from In Vivo Model

#### Effect of BD-8 in Paw Edema

**Figure 9:**
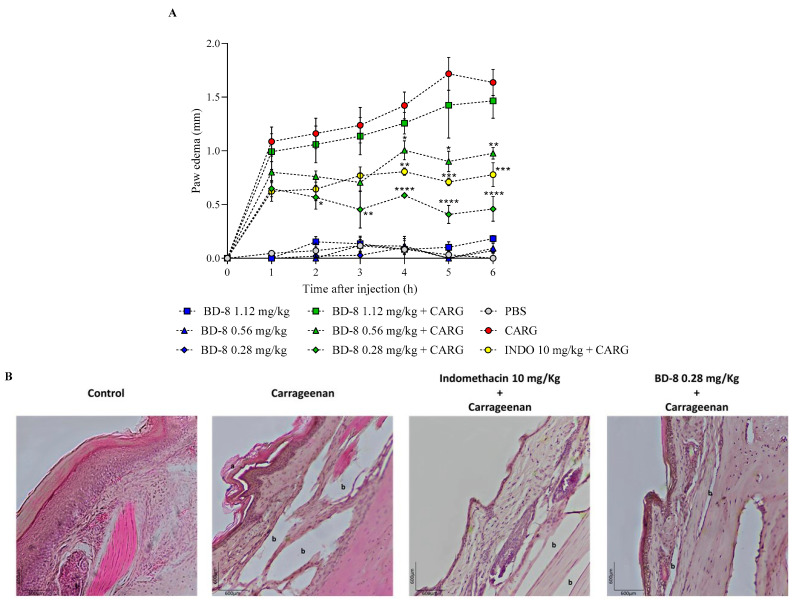
Effect of BD-8 in paw edema. BD-8 dose-response curve (**A**). Histological examination of paw tissue (**B**). Mice were pretreated with 1.12 mg/kg, 0.56 mg/kg and 0.28 mg/kg BD-8 for 3 and 1 hour after the last BD-8 treatment, mice received intraplantar injections of carrageenan in 20 μL PBS in the right hindpaw, and 20 μL of PBS in the left hindpaw. Indomethacin (INDO 10 mg/kg) was used as anti-inflammatory control and injected i.p. one hour before intraplantar challenge. Asterisks denote the significance levels compared with carrageenan group. Data were expressed as mean ± S.E.M. and analyzed by software Graphpad Prism using Student’s t-test followed by unpaired test. ****—(*p* < 0.0001); ***—(*p* = 0.001); **—(*p* = 0.01); *—(*p* = 0.05). (a)—Epidermal layer wear; (b)—Edema formation; PBS–Control group; CARG–Carrageenan; INDO–Indomethacin.

Although BD-8 at 1.12 mg/kg did not show an effect, BD-8 at 0.56 mg/kg and 0.28 mg/kg effectively prevented the paw edema induced by carrageenan. The effect of BD-8 at 0.56 mg/kg was most notable at 6 h, reducing paw edema inflammation by 40.5% (**—*p* = 0.001). However, the most significant effect was observed at the lowest dose, BD-8 0.28 mg/kg, at 5 h, with an inflammation reduction of 76.8% (****—*p* < 0.0001) (Figure 9A).

Histological analysis of paw tissue sections showed a decrease in edema (b) in the BD-8 0.28 mg/kg group and standard drug indomethacin at 10 mg/kg. Microscopic photographs of the control stained with hematoxylin and eosin revealed normal paw tissue with no signs of inflammation (Figure 9B). In the carrageenan-stimulated group, the epidermal layer showed wear (Figure 9B (a)) and an increased edema formation (Figure 9B (b)). However, BD-8 0.28 mg/kg and indomethacin 10 mg/kg treated groups showed low inflammation damage and decreased edema formation. Moreover, BD-8 0.28 mg/kg, demonstrated an even greater reduction in the inflammation process compared to indomethacin.

## 4. Discussion

Several reports have demonstrated cardiotonic steroids’ potential as immunomodulatory compounds, and digoxin benzylidene 8 (BD-8) became a novel benzylidene digoxin derivative synthesized by our research group [20,23,24]. The in silico analyses initially reinforced the potential of BD-8 as a novel cardiotonic steroid since it displayed lower toxicity potential. Collectively, these findings demonstrate the potential of bioinformatics (alongside computational biology and artificial intelligence models) as a screening platform for molecules of more promising biomedical potential [42]. Hence, it remains attractive to test if BD-8 is less toxic under other preclinical models of inflammation.

Treatment with BD-8 in peritoneal macrophages, whether stimulated with ZYM or not, was only toxic at the highest concentration. Previous studies have shown that high concentrations of ouabain can induce cell death by inhibiting Na^+^/K^+^-ATPase [13]. This could be associated with the low viability of peritoneal macrophages observed at a BD-8 concentration of 100 µM. However, other studies have shown that nanomolar concentrations of cardiotonic steroids prevent apoptosis [43,44]. The concentrations of 1 nM, 10 nM, 100 nM, 1 µM, and 10 µM did not confer cytotoxicity, corroborating previous studies with ouabain and marinobufagin [45,46].

ZYM is a fungi-derived polysaccharide that induces a robust inflammatory response in macrophages [44,45]. The ZYM-induced inflammatory response initiates when the polysaccharide binds to Toll-like receptor 2 and glucan receptor dectin-1 present on the macrophage surface. These bindings trigger intracellular signal transduction through these receptors, which induces transcription factors that regulate the expression of inflammation-related genes [46,47,48]. One of the initial responses to stimulation with ZYM was nitric oxide (NO) production. Our data demonstrated that BD-8 downregulates ZYM-induced NO production, along with ZYM-induced iNOS expression. The transcription of the iNOS gene ultimately leads to continuous nitric oxide production until its degradation. The iNOS production is regulated by both transcriptional and post-transcriptional mechanisms [49]. Several studies have shown that other cardiotonic steroids also negatively modulate the iNOS transcription in models of joint inflammation in mice treated with frugoside [50].

Macrophages play a crucial role in modulating autoimmune inflammatory diseases by regulating the levels of inflammatory mediators, either increasing or decreasing them. M1 macrophages are prevalent in the pro-inflammatory environment of autoimmune diseases such as Systemic Lupus Erythematosus (SLE), and thus produce cytokines such as IL-1β, interferon (IFN-γ), and IL-6. In contrast, M2 macrophages in SLE promote the production of anti-inflammatory mediators, such as IL-10. Despite high IL-10 concentrations in SLE, it exerts a pro-inflammatory role due to the elevated levels of type-I IFN, which enhances its pro-inflammatory function, leading to a positive feedback loop of pro-inflammatory cytokine production. Furthermore, the phagocytic function of macrophages is impaired in autoimmune diseases, inhibiting the clearance of apoptotic cells [51,52]. Macrophages also act as the first line of defense against pathogens (bacteria, fungi, and fungal particles) and cancer cells by phagocytosis and secreting both pro-inflammatory and antimicrobial mediators [53,54]. Increased phagocytosis activity is associated with increased NO production, the downregulation of NO production correlates with the reduction of phagocytosis activity upon BD-8 treatment. These data corroborate the NO reduction by marinobufagin as previously demonstrated [55]. Oxidative stress caused by ROS plays a fundamental role in the inflammatory process and can activate various transcription factors associated with signaling pathways in the inflammatory process [56]. The BD-15 molecule (another derivative of digoxin) demonstrated a neuroprotective effect by preventing H_2_O_2_ production, lipid peroxidation, and other factors related to oxidative stress damage [57,58]. However, our data showed that the immunomodulatory effect of BD-8 is not related to ROS production, suggesting that BD-8 may not play a role (or may play a different one) in oxidative stress.

Activated macrophages also produce many pro-inflammatory cytokines, such as IL-1β, IL-6, and TNF-α, as part of the inflammatory response. In addition, anti-inflammatory cytokines, such as IL-10, can also be produced to compensate for the pro-inflammatory response and induce homeostasis [59]. The downregulation of ZYM-induced IL-1β production and upregulation of IL-10 upon BD-8 treatment indicate the immunomodulatory profile of BD-8. Studies have shown that other cardiotonic steroids also negatively modulate IL-1β levels in different models of inflammation, such as marinobufagin, as described by Carvalho et al. [16], and ouabain, as described by Leite et al. [7]. As IL-10 is an anti-inflammatory cytokine, the ability to positively modulate this cytokine suggests an anti-inflammatory mechanism. IL-10 can regulate inflammatory processes by restricting the exacerbated inflammatory response, positively modulating innate immunity, and promoting tissue repair mechanisms [60]. The action of IL-10 is dependent on its interaction with its specific receptor (IL-10R), which initiates the JAK/STAT signaling pathway. Activation of the JAK/STAT pathway decreases several pro-inflammatory cytokines [61]. We observed that BD-8 could increase IL-10 levels at the 100 nM concentration. Moreover, follow-up studies could elucidate the signaling pathway mechanism in more detail.

The signaling pathways mediated by MAPK p38 and ERK are involved in various intracellular responses, encoding biological responses, such as inflammation, cell cycle regulation, cell death, cell development, and differentiation [62,63]. Here, BD-8 negatively modulated the MAPK p38 and ERK signaling pathway mechanisms. These findings agree with an independent study carried out with the cardiotonic steroid ouabain on neutrophil signaling and the immune response, showing that ouabain decreased p38 phosphorylation in neutrophils stimulated with ZYM [8]. Our data demonstrated that BD-8 decreased NF-κB p65 levels. This result corroborates a study carried out with bufalin, a cardiotonic steroid shown to inhibit inflammatory activity in lung tissues by inhibiting NF-kB p65 in murine macrophages, thus demonstrating its anti-inflammatory activity [64]. In addition, this reduction in NF-κB levels could be related to the decrease in IL-1β levels observed in this study. The production and release of the cytokine IL-1β is stimulated by the activation of NF-κB [65], and BD-8 may be interfering with inflammasome activation, as indicated by the simultaneous decrease in IL-1β and NF-κB levels [66]. Similarly, in a ZYM-induced peritonitis model, ouabain could reduce IL-1β by inhibiting NF-κB activation [7].

The results demonstrated that BD-8 decreased Akt and mTOR levels, which are responsible for several cellular processes, such as survival cell, differentiation, and proliferation, which are important in inflammatory tissues [67]. The cardiotonic steroids ouabain have been shown to decrease cell growth and migration of glioma U-87MG by inhibiting the signaling pathway Akt/mTOR [68]. Therefore, our data corroborates the negative modulation of the Akt/mTOR signaling pathway by another cardiotonic steroid. The protein kinase Src is widely expressed in different cell types, and its function depends on its location within the cell. Src can be associated with cellular membranes, such as the plasma, perinuclear, and endosomal membranes. Several studies demonstrate that the binding of cardiotonic steroids to the Na^+^/K^+^-ATPase favors the interaction of the pump with Src. This activated kinase leads to subsequent phosphorylation of different tyrosines, regulating processes such as cell growth, proliferation and motility [69,70]. Our data showed that BD-8 is capable of modulating Src, as demonstrated in other studies with the cardiotonic steroid ouabain [71].

**Figure 10:**
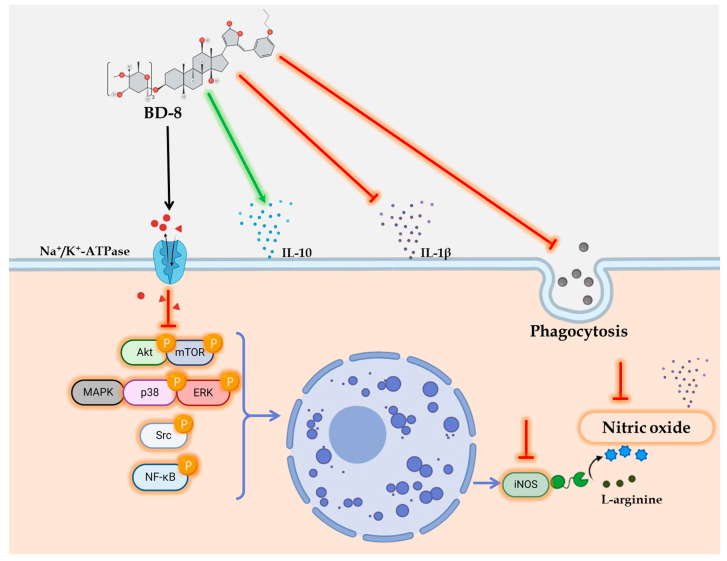
Potential signaling mechanism of the immunomodulatory effect of BD-8. Green arrow—activation signaling; Red arrow with straight tip–inhibition signaling.

Besides the mechanism involving the Na^+^/K^+^-ATPase, cardiotonic steroids have been described as interacting with retinoic acid-related nuclear receptor (ROR)-γ inside the cell [72]. This raises the possibility that the immunomodulatory effect of BD-8 may be partially mediated through ROR-γ modulation, although further studies are needed to elucidate its interaction both in vitro and in vivo. In summary, our data suggest that treatment with BD-8 modulates macrophages immune response, potentially by decreasing phagocytosis processes, nitric oxide, and IL-1β levels through the suppression of the MAPK p38-ERK/NF-κB/Akt/mTOR/Src/iNOS signaling pathways (Figure 10).

## 5. Conclusions

Our data showed the immunomodulatory and anti-inflammatory activity of BD-8 both in vitro and in vivo. These findings contribute to the understanding of the potential role of cardiotonic steroids in inflammation, and further offer an alternative to explore the potential of these molecules under different inflammatory contexts. Finally, more studies are needed to better understand the anti-inflammatory role of BD-8, including in other animal models. The impact of this effect in a different animal model is currently under evaluation.

## Data Availability

The data used to support the findings of this study are available from the corresponding author upon request.

## Supplementary Materials

The following supporting information can be downloaded at: https://www.mdpi.com/article/10.3390/cells13181568/s1, Table S1: Simplified molecular-input line-entry system (SMILES) of cardiotonic molecules compared in this study. BD: γ-benzylidene digoxin derivate; Table S2: In silico predictions of molecule properties of cardiotonic molecules investigated in this study using ProTox3.0. BD: γ-benzylidene digoxin derivate; Table S3: In silico analyses of potential interactions among cardiotonic molecules and signaling pathways using ProTox3.0. BD: γ-benzylidene digoxin derivate; Table S4: In silico analyses of pharmacokinetic and toxicity properties of cardiotonic molecules using Deep-PK. BD: γ-benzylidene digoxin derivate; Table S5: Top ten cell lines predicted by in silico cytotoxicity analysis for four cardiotonic molecules using CLC-pred2.0; Table S6. Cellular pathways predicted in silico to be modulated by four cardiotonic molecules using PASS.
