# Supplementary material for: Evaluation of Anti-Inflammatory Activity of the New Cardiotonic Steroid γ-Benzylidene Digoxin 8 (BD-8) in Mice
Source: Cells. 2024 Sep 18;13(18):1568. doi: 10.3390/cells13181568 (PMC11430542; doi:10.3390/cells13181568)
Supplement: Supplementary file 1 [file cells-13-01568-s001.zip › cells-3161513-supplementary.pdf]

Evaluation of anti-inflammatory activity of the new cardiotonic steroid γ-Benzylidene Digoxin 8 (BD-8) in mice

Davi Azevedo Ferreira, Anna Beatriz Araujo Medeiros, Mariana Mendonça Soares, Éssia de Almeida Lima, Gabriela Carolina Santos Lima de Oliveira, Mateus Bernardo da Silva Leite, Matheus Vieira Machado, José Augusto Ferreira Perez Villar, Leandro Augusto Barbosa, Marcelo Tigre Moura, Sandra Rodrigues-Mascarenhas.

Supplementary Information

Table S1. Simplified molecular-input line-entry system (SMILES) of cardiotonic molecules compared in this study. BD: γ-benzylidene digoxin derivate.

Molecule	SMILES
**BD-8**	[H][C@]9(OC8[C@@H](C)O[C@@]([H])(O[C@]7([H])[C@@H](C)O[C@@]([H])(OC6CC[C@@]1(C)[C@]([H])(CC[C@]4([H])[C@]1([H])C[C@@H](O)[C@]5(C)C(c2cc(=O)oc2=Cc3cccc(OCCCC)c3)CC[C@]45O)C6)C[C@@H]7O)C[C@@H]8O)C[C@H](O)[C@H](O)[C@@H](C)O9
**BD-21**	O=C(C=C1C(CC2)[C@](C)([C@@]2(O)[C@H](CC2)[C@H]3[C@@](C)(CC4)[C@H]2C[C@H]4O[C@H](O[C@H](C)[C@H]2O[C@@H](C[C@H](O)C4O[C@@H](C[C@H](O)[C@@H]5O)O[C@@H]5C)O[C@@H]4C)C[C@@H]2O)[C@H](O)C3)O/C1=C\C(=CC=C1)C=C1
**Digoxin**	C[C@@H]1[C@H]([C@H](C[C@@H](O1)O[C@@H]2[C@H](O[C@H](C[C@@H]2O)O[C@@H]3[C@H](O[C@H](C[C@@H]3O)O[C@H]4CC[C@]5([C@@H](C4)CC[C@@H]6[C@@H]5C[C@H]([C@]7([C@@]6(CC[C@@H]7C8=CC(=O)OC8)O)C)O)C)C)C)O)O
**Ouabain**	C[C@H]1[C@@H]([C@H]([C@H]([C@@H](O1)O[C@H]2C[C@H]([C@@]3([C@@H]4[C@@H](CC[C@@]3(C2)O)[C@]5(CC[C@@H]([C@]5(C[C@H]4O)C)C6=CC(=O)OC6)O)CO)O)O)O)O

Table S2. ***In silico*** **predictions of molecule properties of cardiotonic molecules investigated in this study using ProTox3.0.** BD: γ-benzylidene digoxin derivate.

Molecule	BD-8	BD-21	Digoxin	Ouabain
Molecular weight	941.15	869.05	780.94	584.65
Number of hydrogen bond acceptors	15	14	14	12
Number of hydrogen bond donors	6	6	6	8
Number of atoms	67	62	55	41
Number of bonds	75	70	62	46
Number of rotable bonds	12	8	7	4
Molecular refractivity	246.62	225.71	196.10	140.66
Topological Polar Surface Area	212.29	203.06	203.06	206.60
Octanol/water partition coefficient (logP)	5.44	4.26	2.22	-1.52

Table S3. ***In silico*** **analyses of potential interactions among cardiotonic molecules and signaling pathways using ProTox3.0.** BD: γ-benzylidene digoxin derivate. High probabilities (>0.70) in green were predicted as inactive and in red as active.

Signaling Pathway		BD-8	BD-21	Digoxin	Ouabain
Nuclear receptor signaling	Aryl hydrocarbon Receptor (AhR)	0.95	0.98	1.00	1.00
	Androgen Receptor (AR)	0.87	0.94	0.90	0.96
	Androgen Receptor Ligand Binding Domain (AR-LBD)	0.82	0.82	1.00	1.00
	Aromatase	0.70	0.77	1.00	1.00
	Estrogen Receptor Alpha (ER)	0.57	0.70	0.99	0.76
	Estrogen Receptor Ligand Binding Domain (ER-LBD)	0.99	0.94	0.99	0.99
	Peroxisome Proliferator Activated Receptor Gamma (PPAR-Gamma)	0.52	0.50	0.57	0.92
Stress response pathways	Nuclear factor (erythroid-derived 2)-like 2/antioxidant responsive element (nrf2/ARE)	0.87	0.94	0.97	0.99
	Heat shock factor response element (HSE)	0.87	0.94	0.97	0.99
	Mitochondrial Membrane Potential (MMP)	0.83	0.89	1.00	1.00
	Phosphoprotein (Tumor Supressor) p53	0.58	0.59	0.76	0.66
	ATPase family AAA domain-containing protein 5 (ATAD5)	0.88	0.87	0.85	0.76
Molecular initiating events	Thyroid hormone receptor alpha (THRα)	0.90	0.90	0.90	0.90
	Thyroid hormone receptor beta (THRβ)	0.78	0.78	0.78	0.78
	Transtyretrin (TTR)	0.97	0.97	0.97	0.97
	Ryanodine receptor (RYR)	0.98	0.98	0.98	0.98
	GABA receptor (GABAR)	0.96	0.96	0.96	0.96
	Glutamate N-methyl-D-aspartate receptor (NMDAR)	0.92	0.92	0.92	0.92
	alpha-amino-3-hydroxy-5-methyl-4-isoxazolepropionate receptor (AMPAR)	0.97	0.97	0.97	0.97
	Kainate receptor (KAR)	0.99	0.99	0.99	0.99
	Achetylcholinesterase (AChE)	0.94	0.87	0.98	0.98
	Constitutive androstane receptor (CAR)	0.98	0.98	0.98	0.98
	Pregnane X receptor (PXR)	0.92	0.92	0.92	0.92
	NADH-quinone oxidoreductase (NADHOX)	0.97	0.97	0.97	0.97
	Voltage gated sodium channel (VGSC)	0.95	0.95	0.95	0.95
	Na+/I- symporter (NIS)	0.98	0.98	0.98	0.98
Metabolism	Cytochrome CYP1A2	0.87	0.96	0.99	1.00
	Cytochrome CYP2C19	0.89	0.94	0.99	1.00
	Cytochrome CYP2C9	0.75	0.79	0.92	0.97
	Cytochrome CYP2D6	0.77	0.85	0.78	0.60
	Cytochrome CYP3A4	0.80	0.80	1.00	1.00
	Cytochrome CYP2E1	0.99	0.99	0.99	0.98

Table S4. ***In silico*** **analyses of pharmacokinetic and toxicity properties of cardiotonic molecules using Deep-PK.** BD: γ-benzylidene digoxin derivate. ADMET: absorption. distribution. metabolism. excretion. and toxicity. CNS: Central nervous system. Red: low confidence. Yellow: medium confidence. Green: high confidence. BA: bioavailable. NBA: non-bioavailable. NP: non-penetrable.

**ADMET Predictions**		**BD-8**	**BD-21**	**Digoxin**	**Ouabain**
Absortion	Human Oral (Bioavailability 20%)	BA	BA	NBA	NBA
	Human Intestinal Absortion	Yes	Yes	Yes	Yes
	Human Oral (Bioavailability 50%)	BA	BA	BA	BA
Distribution	Blood-Brain Barrier (CNS)	-2.85	-3.07	-3.43	-3.94
	Blood-Brain Barrier	NP	NP	NP	NP
	Fraction Unbound (Human)	0.68	0.74	0.49	0.73
	Plasma Protein Binding	96.39	95.35	87.26	73.08
	Steady State/Volume of Distribution	1.06	0.96	0.52	0.11
Metabolism	CYP1A2 Substrate	No	No	No	No
	CYP2C19 Substrate	No	No	No	No
	CYP2C9 Substrate	No	No	No	No
	CYP2D6 Substrate	No	No	No	No
	CYP3A4 Substrate	Substrate	Substrate	Substrate	Substrate
Excretion	Clearance	3.35	2.57	0.96	-0.45
	Half-Life	< 3h	< 3h	< 3h	< 3h
Toxicity	Carcinogenesis	Safe	Safe	Safe	Safe
	Liver Injury I	Safe	Toxic	Toxic	Toxic
	Liver Injury II	Toxic	Safe	Safe	Toxic
	Maximum Dose Tolerated	-2.31	-2.49	-3.73	-1.20
	hERG Blockers	Toxic	Toxic	Toxic	Toxic
	Aryl hydrocarbon Receptor (AhR)	Safe	Safe	Safe	Safe
	Androgen Receptor (AR)	Safe	Safe	Safe	Safe
	Androgen Receptor Ligand Binding Domain (AR-LBD)	Toxic	Toxic	Toxic	Toxic
	NR-Aromatase	Toxic	Toxic	Toxic	Toxic
	Estrogen Receptor (ER)	Toxic	Safe	Toxic	Safe
	Estrogen Receptor Ligand Binding Domain (ER-LBD)	Safe	Safe	Safe	Safe
	Glucocorticoid receptor (GR)	Toxic	Toxic	Toxic	Safe
	Peroxisome Proliferator Activated Receptor Gamma (PPAR-Gamma)	Safe	Safe	Safe	Safe
	Thyroid Receptor (TR)	Toxic	Toxic	Toxic	Safe
	Rat (Acute)	4.81	4.98	4.72	4.19
	Rat (Chronic Oral)	8.21	3.06	2.89	3.20
	Respiratory Disease	Safe	Toxic	Toxic	Toxic
	Nuclear factor (erythroid-derived 2)-like 2/antioxidant responsive element (nrf2/ARE)	Safe	Safe	Safe	Safe
	ATPase family AAA domain-containing protein 5 (ATAD5)	Safe	Safe	Safe	Safe
	Heat shock factor response element (HSE)	Safe	Safe	Safe	Safe
	Mitochondrial Membrane Potential (MMP)	Toxic	Toxic	Toxic	Toxic
	Phosphoprotein (Tumor Supressor) p53	Toxic	Toxic	Toxic	Safe

Table S5. Top ten cell lines predicted by *in silico* cytotoxicity analysis for four cardiotonic molecules using CLC-pred2.0. Probability of activation/inhibition. BD: γ-benzylidene digoxin derivate.

Cell Lines (Alphabetical order)	Probability of Activation/Inhibition			
	**BD-8**	**BD-21**	**Digoxin**	**Ouabain**
A549	***	***	0.725/0.020	0.867/0.007
Calu-1	0.557/0.004	0.583/0.003	***	0.617/0.002
HT-1080	***	0.548/0.006	0.699/0.004	0.704/0.004
Kasumi-1	0.481/0.010	0.516/0.005	***	0.631/0.004
M19-MEL	0.608/0.008	0.658/0.004	0.687/0.004	0.604/0.008
MCF7	0.578/0.058	0.588/0.055	0.775/0.015	0.825/0.011
MRC5	***	***	***	0.655/0.006
OVCAR-5	0.489/0.023	0.515/0.020	***	***
PC-3	0.620/0.019	0.639/0.017	0.733/0.009	0.807/0.005
SK-MEL-28	***	***	***	0.759/0.004
SW1990	0.624/0.004	0.664/0.003	0.723/0.002	0.709/0.003
TK-10	0.572/0.014	0.602/0.012	0.687/0.006	***
U-251	***	***	0.660/0.009	***
UACC-62	0.582/0.009	0.612/0.008	0.883/0.003	***
UO-31	0.506/0.018	***	0.724/0.005	***

Table S6. Cellular pathways predicted *in silico* to be modulated by four cardiotonic molecules using PASS. Probability of activation threshold was >0.7. Data was shown as activation/inhibition. BD: γ-benzylidene digoxin derivate.

Cellular Pathways (Alphabetical order)	Probability of Activation/Inhibition			
	**BD-8**	**BD-21**	**Digoxin**	**Ouabain**
4-Nitrophenylphosphatase inhibitor	***	***	0.749/0.003	***
Alkenylglycerophosphocholine hydrolase inhibitor	0.901/0.005	0.809/0.017	0.837/0.013	***
Alkenylglycerophosphoethanolamine hydrolase inhibitor	0.701/0.007	***	***	***
Anesthetic general	0.966/0.002	0.949/0.002	***	***
Antineoplastic	0.811/0.010	0.873/0.005	0.898/0.005	0,878/0,005
	***	***	***	0,717/0,005
Antineoplastic (Lung cancer)	***	***	0.750/0.005	0,773/0,005
Antiprotozoal (Leishmania)	0.789/0.005	***	0.832/0.004	***
Apoptosis agonist	0.707/0.014	0.785/0.009	0.825/0.006	0,788/0,009
Cardiotonic	0.832/0.004	0.865/0.004	0.942/0.003	0,911/0,004
Caspase 3 stimulant	0.852/0.004	0.891/0.004	0.969/0.002	***
Caspase 8 stimulant	***	***	0.722/0.003	***
CDP-glycerol glycerophosphotransferase inhibitor	0.713/0.051	0.767/0.037	0.789/0.032	0,873/0,015

Chemopreventive	0.763/0.005	0.795/0.004	0.833/0.003	0,812/0,004
Cholesterol antagonist	0.777/0.005	***	0.722/0.007	***
CYP2B6 substrate	***	***	0.713/0.012	***
CYP2H substrate	0.810/0.016	0.861/0.009	0.863/0.009	0,829/0,013
CYP3A substrate	0.912/0.005	0.916/0.004	0.950/0.003	0,887/0,006
CYP3A1 substrate	***	***	0.725/0.008	
CYP3A2 substrate	0.970/0.001	0.971/0.001	0.977/0.001	0,920/0,002
CYP3A4 substrate	0.858/0.009	0.862/0.009	0.936/0.004	0,841/0,010
CYP3A5 substrate	***	***	0.718/0.013	***
Diuretic	***	***	0.867/0.002	***
General pump inhibitor	0.743/0.005	0.766/0.004	0.785/0.004	***
Glyceryl-ether monooxygenase inhibitor	0.783/0.006	0.776/0.006	0.799/0.005	0,759/0,007
Hypoxia inducible factor 1 alpha inhibitor	***	***	***	0,914/0,001
Immunosuppressant	***	***	0.742/0.012	0,770/0,009
Membrane integrity antagonist	0.913/0.002	0.866/0.004	0.860/0.004	***
Na+ K+ transporting ATPase inhibitor	***	***	0.943/0.000	***
P-glycoprotein substrate	***	0.794/0.004	***	0,755/0,004
Proliferative diseases treatment	0.798/0.004	0.813/0.003	0.920/0.002	0,967/0,001
Respiratory analeptic	***	***	0.756/0.011	0,754/0,011
UDP-glucuronosyltransferase substrate	0.929/0.003	0.946/0.002	0.991/0.001	0,950/0,002
UGT1A substrate	***	***	0.813/0.004	0,794/0,005
UGT1A1 substrate	***	***	0.813/0.003	***

****
